# Innovative Ink-Based 3D Hydrogel Bioprinted Formulations for Tissue Engineering Applications

**DOI:** 10.3390/gels10120831

**Published:** 2024-12-17

**Authors:** Ana Catarina Sousa, Grace Mcdermott, Fraser Shields, Rui Alvites, Bruna Lopes, Patrícia Sousa, Alícia Moreira, André Coelho, José Domingos Santos, Luís Atayde, Nuno Alves, Stephen M. Richardson, Marco Domingos, Ana Colette Maurício

**Affiliations:** 1 Departamento de Clínicas Veterinárias, Instituto de Ciências Biomédicas de Abel Salazar (ICBAS), Universidade do Porto (UP), Rua de Jorge Viterbo Ferreira, n° 228, 4050-313 Porto, Portugal; anacatarinasoaressousa@hotmail.com (A.C.S.); ruialvites@hotmail.com (R.A.); bilopes@icbas.up.pt (B.L.); pfrfs_10@hotmail.com (P.S.); alicia.moreira.1998@gmail.com (A.M.); andrefmc17@gmail.com (A.C.); ataydelm@gmail.com (L.A.); 2 Centro de Estudos de Ciência Animal (CECA), Instituto de Ciências, Tecnologias e Agroambiente da Universidade do Porto (ICETA), Rua D. Manuel II, Apartado 55142, 4051-401 Porto, Portugal; 3 Associate Laboratory for Animal and Veterinary Science (AL4AnimalS), 1300-477 Lisboa, Portugal; 4 Department of Cell Matrix Biology & Regenerative Medicine, Faculty of Biology, Medicine & Health, The University of Manchester, Manchester M13 9PL, UK; grace.mcdermott@postgrad.manchester.ac.uk (G.M.); fraser.shields@postgrad.manchester.ac.uk (F.S.); s.richardson@manchester.ac.uk (S.M.R.); 5 Department of Animal and Veterinary Sciences, University Institute of Health Sciences (IUCS), Cooperative of Polytechnic and University Higher Education, CRL (CESPU), Avenida Central de Gandra 1317, 4585-116 Paredes, Portugal; 6 Associated Laboratory for Green Chemistry (REQUIMTE-LAQV), Departamento de Engenharia Metalúrgica e Materiais, Faculdade de Engenharia, Universidade do Porto, Rua Dr. Roberto Frias, 4200-465 Porto, Portugal; jdsantos@fe.up.pt; 7 Centre for Rapid and Sustainable Product Development (CDRSP), Polytechnic Institute of Leiria, 2430-028 Marinha Grande, Portugal; nuno.alves@ipleiria.pt; 8 Department of Mechanical and Aerospace Engineering, School of Engineering, Faculty of Science and Engineering & Henry Royce Institute, The University of Manchester, Manchester M13 9PL, UK; marco.domingos@manchester.ac.uk

**Keywords:** alginate, bioink, bioprinting, bone regeneration, collagen, hydroxyapatite, human bone marrow stem/stromal cells

## Abstract

Three-dimensional (3D) models with improved biomimicry are essential to reduce animal experimentation and drive innovation in tissue engineering. In this study, we investigate the use of alginate-based materials as polymeric inks for 3D bioprinting of osteogenic models using human bone marrow stem/stromal cells (hBMSCs). A composite bioink incorporating alginate, nano-hydroxyapatite (nHA), type I collagen (Col) and hBMSCs was developed and for extrusion-based printing. Rheological tests performed on crosslinked hydrogels confirm the formation of solid-like structures, consistently indicating a superior storage modulus in relation to the loss modulus. The swelling behavior analysis showed that the addition of Col and nHA into an alginate matrix can enhance the swelling rate of the resulting composite hydrogels, which maximizes cell proliferation within the structure. The LIVE/DEAD assay outcomes demonstrate that the inclusion of nHA and Col did not detrimentally affect the viability of hBMSCs over seven days post-printing. PrestoBlue^TM^ revealed a higher hBMSCs viability in the alginate-nHA-Col hydrogel compared to the remaining groups. Gene expression analysis revealed that alginate-nHA-col bioink favored a higher expression of osteogenic markers, including secreted phosphoprotein-1 (SPP1) and collagen type 1 alpha 2 chain (COL1A2) in hBMSCs after 14 days, indicating the pro-osteogenic differentiation potential of the hydrogel. This study demonstrates that the incorporation of nHA and Col into alginate enhances osteogenic potential and therefore provides a bioprinted model to systematically study osteogenesis and the early stages of tissue maturation in vitro.

## 1. Introduction

Bone diseases are an important global public health problem that have significant economic effects, particularly for people suffering from osteoporosis [1,2,3]. Research models that replicate the bone structure, its functions, and the cell–cell and cell–matrix interactions that occur in vivo have been developed to ensure the clinical translation of new treatments to more reliably treat these bone pathologies [4]. Considering the ever-increasing growth of bone tissue engineering and the challenge of bone’s complexity, in the last decade, several three-dimensional (3D) models have been developed to mimic the bone microenvironment, aiming to reduce or replace the dependence on animal experiments [5]. Nevertheless, these models are unable to fully replicate the complex structural and cellular composition of bone tissue [6].

Bioprinting has emerged as a transformative approach within regenerative medicine and tissue engineering, demonstrating significant potential for the generation of three-dimensional tissue constructs [7]. A noteworthy application of this technology is in the field of bone tissue regeneration, which has been increasingly emphasized. This is mainly due to the structural and biological complexities that define the nature of bone tissue [8]. The production of 3D models of bone tissue remains a major challenge to achieve, since the selection of materials and manufacturing processes and the establishment of optimal conditions to support multiple cell populations, as well as their osteodifferentiation, leads to the development of these models not being a trivial task [9]. The choice of biocompatible materials plays an important role not only in mimicking the physical properties of bone, but also in providing the biochemical pathways necessary to guide the behavior of stem cells. Various biomaterials, such as calcium phosphate ceramics, bioactive glass, and hydrogels (e.g., alginate), have been used in bioprinting to promote osteogenesis.

Alginate, an FDA-approved natural polysaccharide, is widely employed in biomedical applications as a bioink [10,11]. This widespread use is attributed to its low cost [12], accessibility [13], and ease of preparation [14]. Consequently, alginate-based hydrogels emerge as a promising candidate capable of offering a three-dimensional (3D) microenvironment encompassing both organic and inorganic elements vital for osteoblast functionality. Nevertheless, alginate is an inert material and lacks osteoinductive signals necessary for promoting osteoblast differentiation and bone formation. To address these limitations, additional modifications are required to enhance its osteogenic potential. Combining alginate with collagen (Col), the primary structural component of the extracellular matrix (ECM), and bioactive ceramics, such as hydroxyapatite (HA), can significantly increase osteoconductivity [12,15,16]. These materials were selected due to bone’s hierarchical structure, consisting of living cells immersed in a matrix primarily composed of Col and HA [17]. Researchers focus on mimicking the bone’s composition to achieve a high degree of similarity between the 3D printed scaffold and bone tissue. Col represents nearly 80% of the organic material in bones [18,19]. It is a suitable biomaterial due to its outstanding biocompatibility, degradability, adhesion characteristics, osteogenic induction properties, and minimal immunogenicity [20]. Additionally, nano-HA (nHA) presents stability, biocompatibility, hydrophilicity, and degradability. This ceramic enhances the adhesion and proliferation of osteoblasts and possesses the capacity to establish chemical bonds with the surrounding bone tissue [21]. The combination of alginate, a biocompatible polymer capable of forming stable gels, nHA, an osteoconductive element, and Col, a primary organic component of natural bone, has been demonstrated to improve the biomineralization, promoting natural bone regeneration [22]. These biomaterials have been utilized in bone bioprinting applications to replicate the complex hierarchical structure of bone tissue, which is essential for its biological functions [23,24,25,26]. Hassani et al. developed cell-laden alginate-nHA-Col microcapsules for modular bone tissue, and the results suggest that this structure promotes mineralization and the osteogenesis signaling pathways of the encapsulated cells [27]. Despite their effectiveness in creating controlled microenvironments for osteogenesis, microcapsules present certain limitations. They are unable to fully replicate the complex hierarchical structure of native bone tissue, as cell–material interactions are restricted by the encapsulation process within the alginate matrix. This confinement limits the degree of cell–matrix interaction, potentially hindering cellular behaviors such as migration and differentiation. Additionally, microcapsules present challenges in reproducibility, as their size, composition, and cell distribution can differ. In contrast, bioprinting techniques using the same materials offer advantages in creating more structured, complex 3D environments that more closely mimic native bone architecture. These methods enable enhanced cell–matrix interactions, which can improve osteogenesis. Furthermore, the automation and standardization of biofabrication processes provides greater reproducibility of 3D tissue models.

In the field of regenerative medicine, the integration of biomaterials with mesenchymal stem/stromal cells (MSCs) has been prevalent, as this approach promotes the proliferation and differentiation of MSCs [28,29]. These cells can be isolated from several tissues, including bone marrow [30]. Human MSCs derived from bone marrow (hBMSCs) are considered multipotent stromal cells, and they possess the inherent capacity to differentiate into osteoblasts and form bone tissue, allowing their osteogenic differentiation to be studied directly in a 3D environment [31,32].

In light of these considerations, the present study aimed to develop a bioink formulation tailored for extrusion-based bioprinting, incorporating alginate, nHA, and Col, along with hBMSCs. This is the first study applying these biomaterials and hBMSCs, through biofabrication (using a pressure-assisted extrusion technique), seeking to address the fundamental challenge of replicating the composition of the native ECM in terms of materials (organic and inorganic) to study osteogenesis in 3D. Therefore, this technique ensures the spatial distribution and cell loading efficiency, facilitates the detailed study of cell–biomaterial interactions, and results in the creation of highly structured and functional tissue constructs. This approach provides a bioprinted model to study osteogenesis and the early stages of tissue maturation in vitro. Therefore, the current work highlights the production of a tailored bioink and underlines its significant contribution to the development of a 3D model for studying the osteogenic differentiation of MSCs. The methodology presented in this study can be readily adapted for broader applications in personalized medicine, including 5D bioprinting, which integrates 3D model construction with data on physiological activity, providing a powerful tool for personalized therapies, as demonstrated in previous studies [33,34]. Moreover, this alginate-based system holds potential for promising applications in toxicological testing using organoid models. This flexibility highlights the adaptability of our biofabrication approach, extending its utility to the broader contexts of 5D personalized medicine and organoid-based toxicological screening.

## 2. Results and Discussion

The aim of the study was to explore the combination of alginate, nHA, and Col to create a composite ink for 3D bioprinting of constructs capable of replicating the composition of native bone ECM, facilitating in vitro studies on osteogenesis and contributing to reducing animal experimentation in the field of tissue engineering.

Alginate was used due to its properties, namely, easy adjustability, low price, and accessibility [12,14]. Furthermore, the inclusion of nHA, a mineral, and Col, a protein, has been implemented due to their well-known capacity to enhance osteogenic potential and accelerate the regeneration of modular bone tissue [35]. While the inclusion of hydroxyapatite in hydrogels can improve the osteogenic and rheological properties, Col can support high cell viability, proliferation, and differentiation, providing an optimal microenvironment conducive to cell survival and functional performance of the printed composite [19,36,37].

The concentrations of alginate, nHA, and Col used in this study (2%, 0.5%, and 0.5%, respectively) were selected based on findings from previous research [38]. A 2% alginate concentration was selected for its ideal balance of gel strength and printability, providing excellent gelling properties, structural integrity, and effective cell encapsulation and nutrient diffusion [38]. Additionally, the incorporation of 0.5% nHA and 0.5% Col is based on the research conducted by Hassani et al. [27], which demonstrated that these concentrations contribute to an osteogenic microenvironment favorable to the development of bone tissue. These concentration ratios were chosen to leverage their synergistic effects, aiming to enhance the osteogenic differentiation and the overall structural integrity of the hydrogel [27]. The selected concentrations are consistent with those used in similar studies, but preliminary experiments were conducted to test a range of concentrations for each component. The final concentrations achieved the best balance of printability, structural stability, and biological functionality, as verified by our data.

The dual crosslinking methodology employed in this study follows a sequential process aimed at optimizing both the biological and mechanical properties of the hydrogel. Initially, Col fibrillogenesis was induced by raising the temperature to 37 °C for 90 min, allowing the collagen component to self-assemble into fibrils that closely mimic the natural extracellular matrix. This step is key for promoting cell–matrix interactions, which support cellular adhesion and osteogenic differentiation. Afterwards, CaCl_2_ solution was introduced to initiate the ionic crosslinking of alginate. Despite the rapid gelation of alginate upon contact with Ca^2+^ ions, the solution was left to interact for an extended period of 40 min. This duration was selected to ensure comprehensive crosslinking throughout the hydrogel matrix, thereby enhancing its mechanical stability and functional integrity. The sequential approach of collagen self-assembly followed by alginate crosslinking is essential for creating a composite hydrogel that balances bioactivity with mechanical strength [39]. The dual crosslinking methodology employed presents notable benefits, but it also has limitations, including potential spatial heterogeneity and extended processing times. Future research should aim to address these issues by improving the distribution of crosslinking agents, investigating more efficient methods, and optimizing cell encapsulation techniques to enhance the methodology’s applicability and performance in tissue engineering [39,40,41,42].

### 2.1. Rheological Characterization

**Figure 1:**
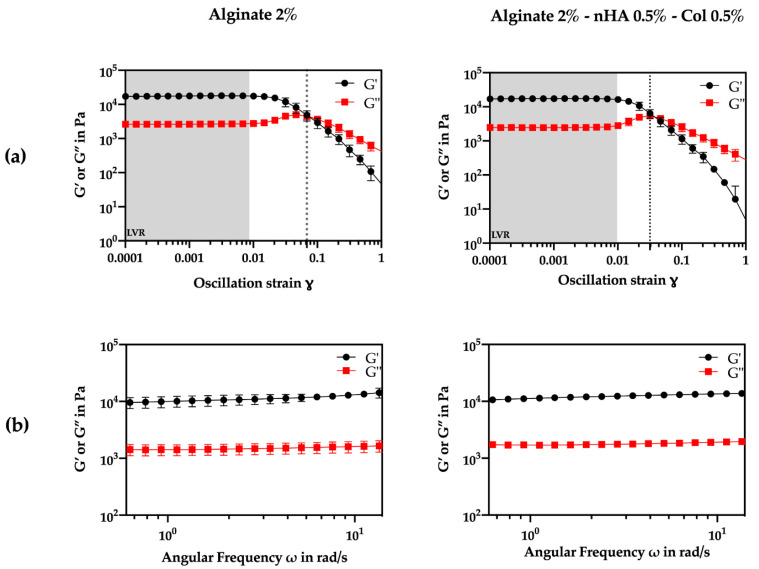
Rheological experiments with the storage moduli (G′) (shown in black) and loss moduli (G″) (shown in red) of crosslinked alginate and alginate-nHA-Col under a range of conditions. (**a**) Amplitude sweeps performed at a frequency of 1 Hz. (**b**) Frequency sweeps performed at an oscillation strain of 0.1%. The evaluations were performed in triplicate.

To achieve optimal print fidelity, several material properties, including viscoelastic behavior, must be considered to ensure that the bioink flows smoothly through the nozzle, maintaining high cell viability during deposition [43]. The viscoelastic characteristics of the crosslinked hydrogels were assessed through oscillatory rheology using amplitude and frequency sweep tests (Figure 1). First, the upper limit of the linear viscoelastic region (LVR) was determined from the storage modulus (G′) curve assuming a ±5% deviation between two consecutive measurements at 0.1% oscillation strain (Figure 1a).

Amplitude sweep tests (Figure 1a) also demonstrated that both formulations exhibited a gel-like or solid structure behavior in the LVR with a higher G′ compared to the G″ (G′ > G″). According to the literature, viscoelastic solids present a higher storage modulus than loss modulus, due to the strong internal chemical bonds and physicochemical reactions in the material [44]. Considering constant amplitude and frequency conditions (1% strain, 1 Hz frequency), the G′ was about ten times higher than the G″, indicating a solid elastic behavior for both formulations. Furthermore, the addition of nHA and Col did not appear to significantly alter LVR (*p* > 0.05). Clearly from the results, nHA and Col did not seem to affect the initial characteristics of the hydrogel. The overall modulus of elasticity and loss values did not change between the gels.

Analyzing Figure 1a also shows that the alginate-nHA-Col group presented a higher yield point (0.00974) compared to the alginate group (0.00864), suggesting that the addition of nHA and Col increased the material’s resistance to initial deformation. Meanwhile, the flow point, indicating the transition to a viscous-dominated behavior, was lower in the alginate-nHA-Col group (0.0318) than in the alginate group (0.0685). These data suggest that although the composite hydrogel requires slightly more stress to begin yielding, it transitions to a flow state more readily, possibly due to the combination of the effects of the nHA reinforcement and the elastic behavior of the Col. To further evaluate the transition from the LVR to the flow state, the flow transition index (ratio of flow point to yield point) was calculated. The alginate group presented a higher index (3.26) compared to the alginate-nHA-Col group (7.93), suggesting that the alginate-nHA-Col group has less tendency to brittle fracture [45].

Frequency sweeps were performed within the linear viscoelastic range (LVR) of both formulations at an oscillation strain of 0.1% (Figure 1b). The results demonstrated that both formulations exhibited viscoelastic gel-like properties, with no clear dependency of the G′ or G″ on frequency. Remarkably, a consistent difference of 8 kPa was observed between G′ and G″ across all tested frequencies for both formulations.

A non-dependence between these values and frequency suggests that the material’s elastic and viscous properties were not influenced by the frequency of the applied stress. This is a characteristic of a gel that is stable and does not change its behavior significantly over a range of frequencies. This type of behavior is typical of gels that are well-formed, and it is often noted in materials that are used in applications where they need to maintain their structure and properties over time [46].

### 2.2. Mechanical Analysis

**Figure 2:**
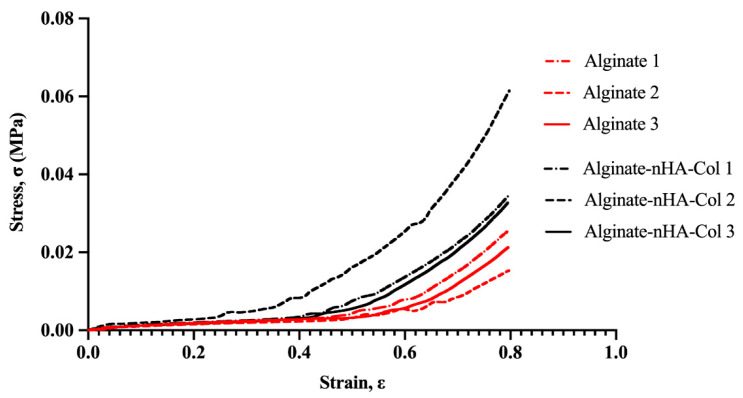
Stress–strain curves resulting from compression tests of alginate hydrogel (red) and alginate-nHA-Col hydrogel (black) (*n* = 3).

The success of a hydrogel is highly dependent on its mechanical properties, as these properties ensure the hydrogel’s structural integrity under both in vitro and in vivo conditions [47]. Parameters such as porosity, pore size, composition, degree of crosslinking, and ionic strength of materials can significantly influence their mechanical strength [48]. In the present work, the mechanical properties of alginate-based gels were evaluated through compressive mechanical testing. Figure 2 illustrates the compressive strength-strain curve and Table 1 provides the Young’s modulus measurements of the alginate-based hydrogels.

Based on the stress–strain curve (Figure 2), the alginate-only hydrogel presented a lower stress–strain behavior due to its brittle nature, whereas the composite hydrogel comprising nHA and Col revealed higher stress–strain behavior due to the combined effects of nHA’s reinforcement and Col’s elasticity. nHA contributed to the reinforcement of the hydrogel matrix, distributing stress more evenly and enhancing the material’s toughness and resistance to deformation [48]. On the other hand, Col, being a fibrous protein, provided elasticity and improved the tensile strength of the hydrogel, providing a more flexible matrix that could stretch further before rupturing [49]. The combination seemed to make the hydrogel more durable and capable of withstanding higher stresses and strains.

Young’s modulus was calculated at 10% strain and the addition of 0.5% (*w*/*v*) nHA and 0.5% (*w*/*v*) of Col 0.5 wt % to alginate decreased the Young’s modulus of Alg hydrogel from 7.33 to 6.80 MPa (Table 1). Notwithstanding, no statistically significant differences were observed in the average Young’s modulus between the two groups.

Based on these findings, the hydrogel composed of only alginate appeared to have a higher initial stiffness (higher Young’s modulus) but a lower capacity to withstand large deformations (lower stress–strain behavior) due to its more fragile nature.

Meanwhile, the hydrogel composed of alginate, nHA, and Col seemed to be more flexible initially (lower Young’s modulus) and better able to withstand large deformations (higher stress–strain behavior) due to the addition of Col, which increased elasticity, and nHA, which reinforced the matrix.

The combination of the different components in the hydrogel resulted in a material that can better absorb and dissipate stresses, resulting in greater deformation capacity and final strength, despite being initially more flexible.

The bioink formulation’s rheological and mechanical properties were tailored to enhance the bioprinting process, allowing mimicry of the complex environment of the bone’s ECM, which is crucial for supporting osteogenesis in vitro.

### 2.3. Hydrogel Swelling Behavior

**Figure 3:**
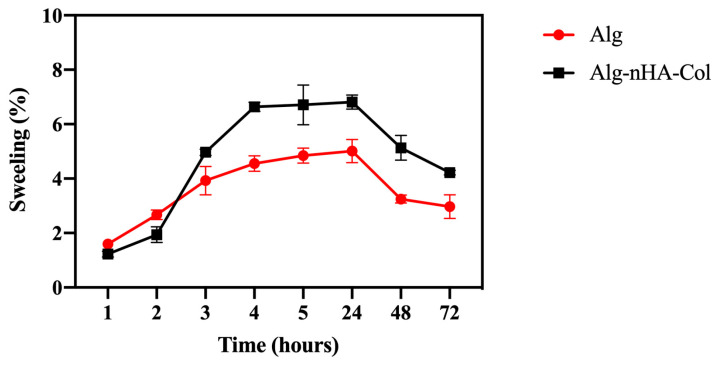
Hydrogel swelling rate from 1 to 72 h. Alg: alginate (red); Alg-nHA-Col (black) (*n* = 3).

The swelling properties play a fundamental role in facilitating the release of molecules, absorption of biofluids, and distribution of nutrients in the structure [35]. Swelling rates are an important feature of bioinks, with ideal formulations undergoing controlled degradation to facilitate new tissue growth [50,51,52,53]. Hydrogels intrinsically swell as solvent molecules infiltrate the spaces within the polymeric network. In 3D bioprinting, the extent of swelling is reflected in the dimensional alterations of bioprinted structures [54,55,56]. Moreover, the swelling ratio is essential in modulating the drug release dynamics from these polymeric systems [56,57,58]. Hydrogel swelling behavior (pH = 7.4 at 37 °C) was evaluated at time intervals of 1, 2, 3, 4, 5, 24, 48, and 72 h. The absorption of DPBS by the hydrogels is shown in Figure 3. The hydrogels appeared to absorb a significant amount of DPBS at first, and then more gradually over time. The hydrogel appeared to reach swelling equilibrium in a relatively short time (3 h). The maximum swelling ratio was noted up to 24 h. The decrease in swelling rate noted after 24 h may result from the hydrogel reaching equilibrium, where osmotic forces balance the polymer network’s elastic forces, leading to reduced swelling. Additionally, hydrolytic degradation of some alginate-based samples under the test conditions may have compromised structural integrity and water retention capacity [59].

Although the data were not significantly different, after 3 h in culture, the hydrogel with nHA and Col seemed to increase the swelling compared to the hydrogel with alginate only. The incorporation of Col and nHA to alginate can increase the swelling rate of the resulting composite material due to the enhanced stability and mechanical strength of the alginate hydrogel, leading to a more controlled degradation rate. The incorporation of Col and nHA into alginate matrices may potentially influence the swelling rate of the resulting composite hydrogels. Col, being a hydrophilic material, presents more hydrophilic bonds, which can result in water retention and consequently affect the swelling behavior [60]. Additionally, the hydrophilic nature of hydroxyapatite in the material composition can lead to overall water absorption and potentially enhance cell growth, proliferation, and viability within the material [21]. For bone mimicry, a high degree of swelling is particularly important, as it allows cell infiltration into the hydrogels and maximizes cell growth within the structure [61].

### 2.4. Bioprinting of Cell-Laden Hydrogels

**Figure 4:**
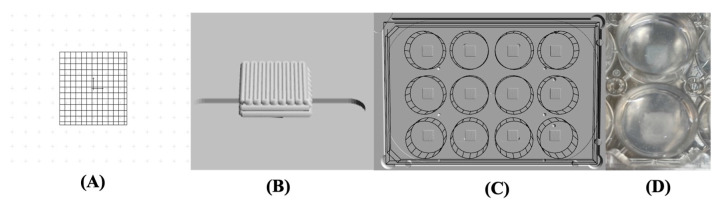
(**A**) CAD model designed in BioCAM™ software (version 2.0); (**B**) 3D generated model; (**C**) 3D generated model in a 12-well plate; (**D**) Cell-laden print in the agarose support bath.

This study integrated bioprinting and pressure-assisted extrusion technologies in the printing protocols for alginate-based bionks. The printing process was carried out using the RegenHU 3D Discovery Evolution system with 2% (*w*/*v*) alginate bioinks. This formulation was selected based on prior studies due to its low viscosity and shear-thinning properties, making it suitable for bioprinting cellular inks [38]. The bioprinting parameters, including needle diameter, pressure, feed speed, filament distance, and thickness, were properly adjusted to establish an optimum equilibrium between printing resolution, speed, and cell activity (Table 2). The printing parameters were adjusted to guarantee high cell viability during the printing process while ensuring the extrusion of accurate filaments, with the external filament diameter corresponding the internal diameter of the nozzle used. This precision is of paramount importance to ensure the fidelity of the structural form in the final constructs, thus achieving an accurate physical replication of the digital CAD model (Figure 4A–C).

Given the low viscosity of the ink formulations used, without compromising shape fidelity [38], suspension bioprinting strategies were employed. A 0.5% (*w*/*v*) agarose support bath was utilized to maintain the structure of the printed material in suspension (Figure 4D). Once the material had been deposited and crosslinked, the suspension bath was removed and the hydrogels with the cells could grow.

### 2.5. Live/Dead Staining

**Figure 5:**
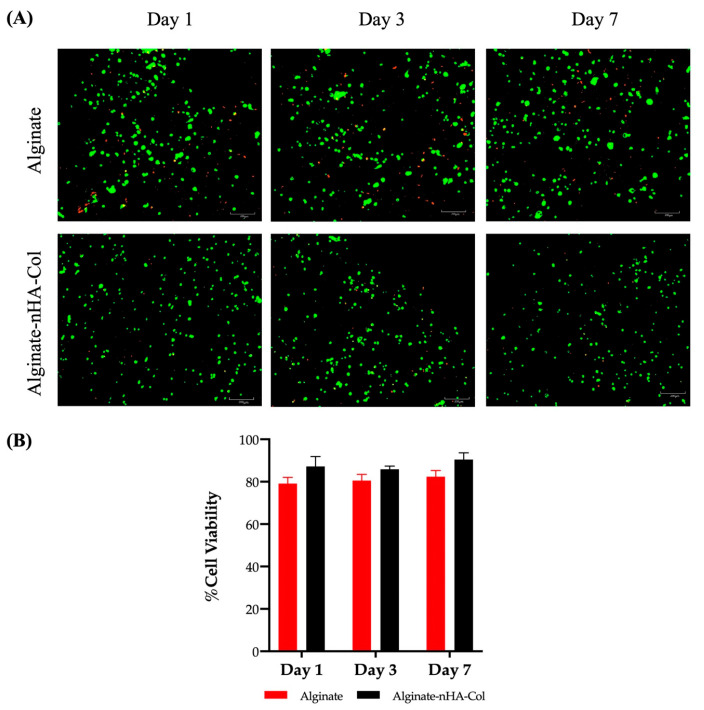
(**A**) Live/dead staining images of printed hBMSCs in alginate and alginate-nHA-Col after 1, 3, and 7 days in culture. Live cells are stained green and dead cells are stained red. (**B**) The percentage of cell viability of bioprinted hBMSCs cultured in 2% alginate and 2% alginate-0.5% nHA-0.5% Col for 1, 3, and 7 days (*n* = 3).

Cell viability must be maintained both during and after the 3D tissue printing process. Therefore, an assessment of cell viability (hBMSCs) was performed using a Live/Dead analysis. This analysis was conducted after 1, 3, and 7 days in culture (Figure 5A,B).

The staining demonstrated high levels of cell viability in both alginate and alginate-nHA-Col hydrogels throughout all time points (Figure 5A).

The hBMSCs viability and distribution did not appear to depend on the inclusion of nHA and Col to the gel, and the cells seemed to be homogeneously distributed in both hydrogels. In fact, some cell death was observed throughout the experiment, although this was to be expected due to exposure to significant shear stress during the printing process, which can cause cell damage and death [62]. Nevertheless, the results of the alginate-nHA-Col group indicated that live cell portions remained above 80% one week after processing (Figure 5B). It is important to note that alginate-nHA-Col group presented slightly higher values of cell viability, but with no significant differences when compared to the alginate group throughout the experiment.

In addition, data showed that a spherical cell morphology was preserved throughout the culture in both conditions. Maintaining a spherical cell morphology within bioinks is advantageous for preserving cell viability and functionality throughout the bioprinting process. This preservation of native cell structure is essential for ensuring proper differentiation into specific cell lineages, which is critical for effective tissue engineering applications [53].

These findings suggest that alginate-nHA-Col hydrogels were non-cytotoxic and provided a supportive matrix conducive to high cell viability of hBMSCs. The results confirmed the hydrogels’ ability to sustain hBMSC culture, suggesting that the pressure-assisted extrusion process did not adversely affect cell viability.

### 2.6. Cell Viability Assay

**Figure 6:**
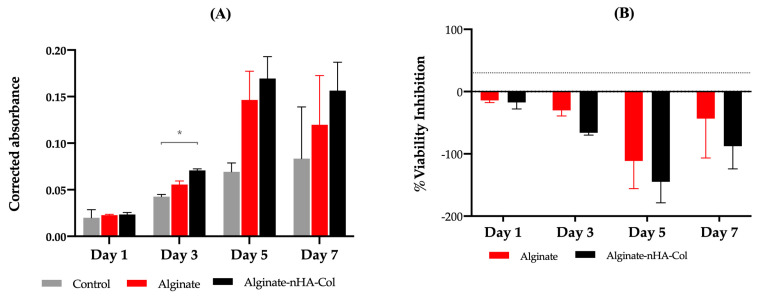
(**A**) Corrected absorbance evaluated by Presto Blue^®^ viability assay for hBMSCs (*n* = 3). Result significances are presented through the symbol (*), according to the *p* value, with * *p* < 0.05. (**B**) Percent viability inhibition assay. The results were normalized in relation to the control. The 30% threshold shown (dashed line) represents the inhibition above which the effect is considered cytotoxic (under ISO 10993-5:2009 guidelines).

In accordance with the ISO 10993-5:2009 guidelines, the assessment of cellular viability was determined using PrestoBlue^TM^ (Thermo Fisher Scientific, Waltham, MA, USA) on both alginate and alginate-nHA-Col hydrogels in the presence of hBMSCs. The control group, devoid of hydrogels, was also included for reference. Figure 6A represents the corrected absorbance values for the designated time points (1, 3, 5, and 7 days). The data showed cell proliferation in all groups up to day 5. While the hydrogels containing alginate, nHA, and Col exhibited slightly higher cell viability rates compared to the other groups, statistically significant differences were only observed on day 3. In contrast, alginate-nHA-Col hydrogels showed significantly greater absorbance than the control group, suggesting enhanced cell adhesion and proliferation. These findings are consistent with previous studies, indicating that the alginate-nHA-Col hydrogels demonstrated superior cytocompatibility overall when compared to the control group [27].

Figure 6B displays the viability inhibition percentages, normalized to the control group. According to the ISO 10993-5:2009 guideline, a cytotoxic effect is considered when an inhibition of viability larger than 30% is identified (outlined in Figure 6B by dashed lines). These findings suggest that both alginate and alginate-nHA-Col can be classified as non-cytotoxic.

In agreement with the results presented in the Live/Dead analysis, the viability results confirmed that, on one hand, the hydrogels are supportive of hBMSCs encapsulation and function and that, on the other, the printing process does not negatively impact cell survival, which remained viable for up to seven days post-encapsulation.

### 2.7. Gene Expression

**Figure 7:**
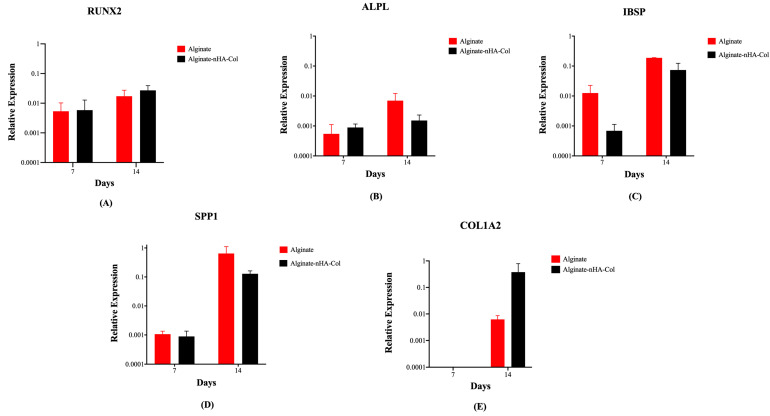
Gene expression levels of printed hBMSCs in alginate and alginate-nHA-Col bioinks after 7 and 14 days under osteogenic differentiation: runt-related transcription factor 2 (RUNX2) (**A**), alkaline phosphatase (ALPL) (**B**), integrin-binding sialoprotein (IBSP) (**C**), secreted phosphoprotein-1 (SPP1) (**D**), and collagen type 1 alpha 2 chain (COL1A2) (**E**).

Osteogenesis of hBMSCs cultured in alginate and alginate-nHA-Col for 7 and 14 days was evaluated using qPCR for a panel of key differentiation marker genes (Figure 7). This gene expression analysis provided insights into the biofunctionality and osteoinductive potential of the inks for bone tissue regeneration.

After 14 days of culture, gene expression analysis revealed a higher expression of osteogenic markers (Figure 7) in all experimental groups compared to the same groups after 7 days. At both time points, the expression of RUNX2 in the alginate-nHA-Col group was slightly higher than the alginate group at both time points (Figure 7A). RUNX2 is a gene expressed during early bone differentiation, and its increased expression from 7 to 14 days is consistent with expected osteogenic progression. Similarly, in the study by Im et al., bioinks composed of alginate, tempo-oxidized cellulose nanofibrils, and polydopamine nanoparticles demonstrated an increase in RUNX2 gene expression over the same time period, further supporting these findings [8]. This gene plays a crucial role in regulating the osteogenic differentiation of hBMSCs and presence of this gene leads to the formation of mature osteoblasts and complete bone formation [63]. ALPL is associated with the biological process of endochondral ossification/osteoblast differentiation [64] and showed a minimal increase from day 7 to day 14 (Figure 7B). Gene expression of IBSP demonstrated an increase in both groups from 7 to 14 days (Figure 7C). IBSP expression has been shown to increase the expression of key osteogenic and angiogenic markers, indicating its beneficial role in promoting the formation and vascularization of bone tissue [65,66]. Furthermore, IBSP encodes bone sialoprotein, a protein involved in matrix production by promoting the nucleation of hydroxyapatite crystals during the mineralization process [67]. This trend was similarly observed in the study by Van der Heide et al., where IBSP expression increased over time, confirming its role in bone mineralization [68].

Although not statistically significant, the expression of SPP1 in the alginate group was higher than in the alginate-nHA-Col group (Figure 7D). SPP1 is a crucial component of the organic matrix of bone and serves as a binding protein for osteoclasts, the cells responsible for bone remodeling [69].

COL1A2 was not expressed in any of the groups after 7 days (Figure 7E). However, after 14 days, the alginate-nHA-Col group showed an up-regulation of COL1A2 expression compared to the alginate group. The observed increase in COL1A2 gene expression aligns with findings reported in the literature [70]. Col is a major ECM protein synthesized by osteoblasts and significantly contributes to bone strength [71]. The observed increased expression of these osteoblastic genes in 3D hBMSCs constructs after 14 days suggests that the incorporation of alginate, nHA, and Col elements in the bioink is important for enhancing osteogenic differentiation.

The presence of bone-related gene expression in alginate-nHa-Col at day 14 also suggests a positive and sustained long-term osteogenic effect in hBMSCs. Nevertheless, osteogenic differentiation is a complex process that involves the sequential expression of several genetic markers at different times. For instance, early markers such as RUNX2 and osterix are expressed during the initial phases of differentiation, while later markers such as SPP1 and osteocalcin are associated with mature osteoblasts and mineralization [72]. As future research, it is important to evaluate genetic markers over an extended period, such as 21 days. In addition, further studies, such as staining with ALP and Alizarin Red, would be pertinent in providing better validation of the support’s functionality and osteogenic differentiation.

## 3. Conclusions

The current work developed a composite hydrogel bioink to replicate native tissue composition for 3D osteogenesis studies, enabling efficient cell distribution, tissue structuring, and MSC differentiation analysis.

Rheological tests revealed that adding nHA and Col maintains viscoelastic properties, forming solid-like structures with a G′ about ten times higher than the G″, confirming solid elastic behavior. Mechanical tests suggest that the incorporation of Col and nHA enhances the hydrogel’s mechanical performance under compressive stresses. Additionally, swelling behavior analysis indicates that the inclusion of Col and nHA into the alginate matrix increases the swelling rate of the composite hydrogels, thereby promoting optimal cell proliferation within the structure.

The LIVE/DEAD^TM^ results of the alginate-nHA-Col group showed that live cell portions remained above 80% one week after processing. Corroborating this, PrestoBlue results also confirm the ability of developed materials to support the encapsulation and printing of hBMSCs without significant loss cell viability one week post-printing. PCR analysis demonstrated that the alginate-nHA-Col enhanced the expression of osteogenic markers, such as SPP1 and COL1A2, in hBMSCs, suggesting the hydrogel’s pro-osteogenic differentiation potential.

Future studies should include measurements of final Young’s modulus, porosity, and structural analysis via Scanning Electron Microscopy (SEM) analysis, along with the characterization of chemical composition and crystalline structure using X-ray diffraction (XRD) analysis, in order to optimize 3D bioprinted models for accurately mimicking bone tissue properties and enhancing their functional relevance in regenerative medicine applications.

The incorporation of nHA and Col into alginate appears to form a bioink with optimum physical and biological properties, providing a promising 3D bioprinted model to mimic bone tissue. This study represents a significant step forward in the development of bioinks for this field, offering a viable and ethical solution for studying osteogenesis and early tissue maturation in vitro.

## 4. Materials and Methods

### 4.1. Materials

Sodium alginate (#180947-100G), nHA (particle size < 200 nm; #677418-25G) and Col type I (#5074-35ML) were purchased from Sigma-Aldrich (Saint Louis, Missouri (MO), United States of America (USA)). The hydrogel formulations were prepared using an agarose (#A6013-100G) fluid gel bath also obtained from Sigma-Aldrich (Saint Louis, MO, USA). Subsequently to their production, the hydrogels were cross-linked using calcium chloride dihydrate (CaCl_2_·2H_2_O; #22317.230-250G), from VWR Chemicals (Bridgeport, PA, USA).

### 4.2. Preparation of Alginate-Based Solutions

**Figure 8:**
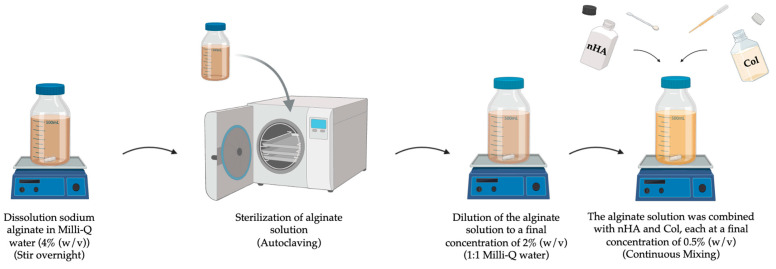
Graphical representation of the preparation of alginate and alginate-nHA-Col-based solutions.

The alginate solution was prepared by dissolving sodium alginate (4% (*w*/*v*)) in Milli-Q water with continuous magnetic stirring overnight. Following this, the 4% alginate solution underwent sterilization via autoclaving and was subsequently subjected to a 1:1 dilution with Milli-Q water, resulting in the formulation of a 2% alginate solution. For the preparation of the alginate-nHA-Col solution, the alginate solution was combined with nHA (0.5% (*w*/*v*) and Col (0.5% (*w*/*v*)) (Figure 8). Table 3 provides an overview of the compositions of the alginate-based solutions.

### 4.3. Cell Culture and Maintenance

All experiments followed the relevant guidelines and were conducted with approvals from the NHS Health Research Authority National Research Ethics Service (21/NS/0056) and the University of Manchester, as well as written informed donor consent. The cells used in this study were previously isolated from bone marrow collected from a 49-year-old female donor after hip replacement surgery. The Strassburg et al. protocol was used to isolate the cells [73]. The hBMSCs were cultured under standard conditions in αMEM media (Sigma-Aldrich, # M4526, Saint Louis, MO, USA) enriched with 10% (*v*/*v*) fetal bovine serum (FBS) (Gibco, Thermo Fisher Scientific, Waltham, MA, USA, #A3160802) 110 mg L^−1^ sodium pyruvate, 1000 mg L^−1^ glucose, 100 U mL^−1^ penicillin, 100 μg mL^−1^ streptomycin (Gibco, #15140122) 0.25 μg mL^−1^ amphotericin (Gibco, #15290026) and 2 mM GlutaMAX (Gibco, #35050061). The hBMSCs were then maintained at 37 °C in an 80% humidified atmosphere with 5% CO_2_.

### 4.4. Oscillatory Rheological Measurements

The Anton Paar RheoCompass™ rheometer (MCR92, Anton Paar, Graz, Austria) operated with Anton Paar RheoCompass v1.30.1164 software was used to evaluate the rheological properties of the samples. Measurements were conducted using a PP12.5 measuring plate, featuring a diameter of 12.5 mm and a 1 mm gap. Alginate-based hydrogels with different compositions (as detailed in Table 4) were prepared following the transwell methodology. Briefly, 400 μL of the solution was pipetted onto 12-well plate transwell inserts. The alginate-nHA-Col hydrogels gelation was achieved through a dual-crosslinking procedure. Initially, Col crosslinking was induced by incubating at 37 °C for 90 min. Subsequently, the alginate was gelled using 150 μM calcium chloride (CaCl_2_) solution in Milli-Q water for 40 min, as the process used for alginate-only hydrogels. Finally, the hydrogels were removed from the inserts and washed with DPBS.

### 4.5. Hydrogel Swelling Behavior

Hydrogel swelling behavior was assessed under physiological conditions (pH = 7.4 at 37 °C). Initially, the weight of the hydrogels (prepared as outlined in Section 4.3) was measured post-crosslinking using precision scales (GR-300-EC, A&D Instruments, Abingdon, UK). Subsequently, these hydrogels were immersed in DPBS at 37 °C within a 12-well plate, and the weight of the swollen hydrogels was recorded. At specific time intervals (1, 2, 3, 4, 24, 48, and 72 h), the hydrogels were reweighed after blotting off excess water with tissue paper. The swelling behavior was quantified as a percentage of the swelling ratio, computed using Equation (1).

Swelling ratio (%) = (Wf − Wi)/Wi × 100,(1)

Wf and Wi are the final and initial weights of the hydrogel (in g), respectively.

### 4.6. Mechanical Analysis

The compressive strength and Young’s modulus (expressed in kPa) of the hydrogels were assessed using the ElectroForce 5500^®^ Test instrument (TA Instruments, New Castle, DE, USA) equipped with a 5 N load cell. Alginate and alginate-nHA-Col hydrogels (prepared as outlined in Section 4.4 of the Section 4) were tested in triplicate, each sample having a height of 3 mm and a diameter of 12 mm. A compression test was conducted employing a flat probe with a diameter of 10 mm and a cross-head displacement rate of 2.0 mm/min. Compression was continued until 80% strain was reached. The compressive (Young’s) modulus was determined by calculating the slope of the linear portion of the stress–strain curve at 10% strain. The settings for the loading procedure were programmed using the WinTest^®^ Software (WinTest 8.0) (TA instruments). The real-time monitoring of the applied load was performed using the WinTest^®^ Software.

### 4.7. Bioprinting of Cell-Laden Hydrogels

**Figure 9:**
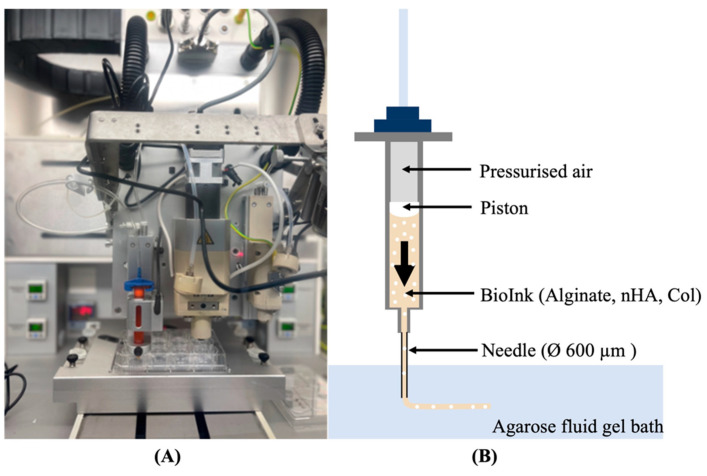
(**A**) 3D Discovery Evolution bioprinter; (**B**) Schematic representation of the pressure-assisted extrusion process, in which pressurized air drives the bioink from the bioprinter cartridge through a needle into a well plate containing a suspension bath.

A pressure-assisted extrusion printer (3D Discovery, RegenHU, Villaz-St-Pierre, Switzerland) with an enclosed biosafety cabinet was employed in the fabrication of cell-laden constructs (Figure 9A). Alginate-based inks loaded with hBMSCs at a density of 2 × 10^6^ cells/mL were prepared as described in Section 4.2 and Section 4.3 and printed inside a suspension bath of agarose particles (Figure 9B) [74], using a set of process parameters previously optimized in our lab. The suspension bath was prepared based on a protocol originally reported by Moxon et al. [75].

Square-based scaffolds (6 × 6 mm) were designed using BioCAD^TM^ software (version 2.0) (RegenHU, Villaz-St-Pierre, Switzerland) and printed in 12-well plates under sterile conditions and at room temperature. Following bioprinting, crosslinking was performed in a two-step process. Initially, Col fibrils were allowed to self-assemble for 90 min at 37 °C. Subsequently, a CaCl_2_ solution was added to the wells and incubated for 40 min at 37 °C to induce the crosslinking of alginate. The cell-laden hydrogels were then rinsed with DPBS and cultured in αMEM medium at 37 °C under 5% CO_2_ and 80% humidity.

### 4.8. Live/Dead Staining

A Live/Dead assay kit (Invitrogen^TM^, Thermo Fisher Scientific, Waltham, MA, USA, #L3224) was used to evaluate the viability of hBMSCs cultured on alginate and alginate-nHA-Col hydrogels. According to the manufacturer’s protocol, 600 µL of assay solution containing 4 µM ethidium homodimer-1 (EthD-1) and 2 µM calcein AM were added to the cell-hydrogel constructs. After a 20 min incubation, the cells were washed with DPBS and imaged using the CellVoyager™ CQ1 Benchtop High-Content Analysis System (Yokogawa, Tokyo, Japan) with an excitation wavelength of 495 nm. Cell imaging was conducted at one, three, and seven days post-culture, with three images acquired per time point. The acquired images were systematically analyzed, and subsequently, the number of cells quantified using ImageJ2 software (version: 2.3.0/1.53q). Cell viability was assessed by calculating the ratio of live cells to the total cell count. To ensure reproducibility, all measurements were conducted in triplicate at each time point.

### 4.9. Cell Viability Assay

To evaluate the cellular viability of hBMSCs within alginate-based hydrogels, the PrestoBlue™ (Invitrogen^TM^ #A13261) assay was performed at one, three, five, and seven days. The evaluation was performed as previously described [21]. In brief, at each timepoint, the culture media was aspirated and substituted with fresh complete media supplemented with 10% (*v*/*v*) of the PrestoBlue^TM^ reagent.

After a one-hour incubation at 37 °C in a controlled environment with 80% humidity and 5% CO_2_, the supernatant media were transferred to a 96-well plate. Absorbance measurements were taken at 570 nm and 595 nm using a SPARK^®^ multimode microplate reader (TECAN, Männedorf, Switzerland). Cell viability was quantified through absorbance spectroscopy by calculating the corrected absorbance, determined by subtracting the absorbance at 595 nm (used for normalization) from that at 570 nm (reflecting the experimental outcome). The resulting values were further adjusted by subtracting the average absorbance of control wells from that of the experimental wells. Residual substances were removed by gently washing the cells with DPBS, and fresh culture medium was added to each well. All measurements were conducted in triplicate at each time point.

### 4.10. Gene Expression

To quantify gene expression levels, RNA extraction and complementary DNA (cDNA) synthesis were performed following established protocols [76,77]. Quantitative real-time polymerase chain reactions (qPCRs) were carried out on a StepOnePlus system (Applied Biosystems, Massachusetts, EUA), utilizing pre-designed intron-spanning primers (Sigma-Aldrich, Saint Louis, MO, USA) (Table 4). In brief, a total of 10 ng cDNA was added to each reaction in addition to 5 μL of Fast SYBR Green Reagent, 1 μL of forward primer, 1 μL of reverse primer, and 2.5 μL of molecular biology grade water.

cDNA synthesized in-house from Total Human RNA (Clontech) served as the positive control in this study. The levels of osteogenic genes were standardized by normalization against the pre-validated reference gene GAPDH [78].

### 4.11. Statistical Analysis

To ensure experimental reproducibility, all experiments were performed in biological triplicates to capture variability between samples, and each assay was conducted in technical triplicates to account for within-experiment variability.

Statistical tests were carried out using GraphPad Prism^®^ (version 10.3.1 for mac OS, La Jolla, California (CA), USA). Results were presented as mean ± standard error of the mean (SE). Comparisons between groups were made using one-way ANOVA, followed by Tukey’s multiple comparisons test. Exclusively for the comparison of gene expression, a Mann–Whitney U test was used. Statistical significance was acknowledged solely when *p* ≤ 0.05. Significance levels were denoted by asterisks (*), with * *p* < 0.05, ** *p* < 0.01, *** *p* < 0.001, and **** *p* < 0.0001.

## Figures and Tables

**Table 1:** Young’s modulus measurement of alginate and alginate-nHA-Col hydrogels produced (mean ± sd values) (*n* = 3).

Parameter (MPa)	Alginate	Alginate-nHA-Col (*w*/*v*)	*p* Value
Young’s Modulus	7.33 ± 1.21	6.80 ± 0.835	0.567

**Table 2:** Parameters used in the RegenHU 3D Discovery Evolution system. The values are shown in the respective units.

Parameters	Values (Unit)
Needle Diameter	600 µm
Pressure	0.02 MPa
Feed Rate	10 mm/s
Filament Distance	500 mm
Thickness	500 mm

**Table 3:** Composition of alginate-based solutions.

Solutions	Sodium Alginate (*w*/*v*)	nHA (*w*/*v*)	Col (*w*/*v*)
Alginate	2	-	-
Alginate-nHA-Col	2	0.5	0.5

**Table 4:** Name, accession number, forward/reverse sequences, and concentrations of the primers used to assess osteogenic differentation.

Primer	Accession Number	Forward Primer Sequence 5′-3′	Reverse Primer Sequence 5′-3′	Concentration (nM)
glyceraldehyde-3-phosphate dehydrogenase (GAPDH)	NM_001256799	CTCCTCTGACTTCAACAG	CGTTGTCATACCAGGAAA	600
runt-related transcription factor 2 (RUNX2)	NM_001024630	CGCTGCAACAAGACC	CGCCATGACAGTAACC	900
alkaline phosphatase (ALPL)	NM_000478	ACGTCTTCACATTTGGTG	GGTAGTTGTTGTGAGCATA	450
integrin-binding sialoprotein (IBSP)	NM_004967	GACTGCTTTAATTTTGCTCAG	GTCACTACTGCCCTGAAC	600
secreted phosphoprotein-1 (SPP1)	NM_001040058	CTGACATCCAGTACCCTG	CAGCTGACTCGTTTCATA	600
collagen type 1 alpha 2 chain (COL1A2)	NM_000089	TGAAGCTGGTCCCCAAGGA	AATACCAGGAGCGCGCCGTTG	300

The measurement conditions were as follows: Amplitude Sweep: Temperature = 25 °C, Frequency = 1 Hz, Oscillation Strain = 0.01–100%; Frequency Sweep: Temperature = 25 °C, Frequency = 0.1–10 Hz, Oscillation Strain = 0.1%. The data were measured in triplicate and the results were shown as mean ± standard deviation.

## Data Availability

Data are contained within the article.

## List of Abbreviations

3D	three-dimensional
ALPL	alkaline phosphatase
CaCl_2_	calcium chloride
Col	collagen
COL1A2	collagen type 1 alpha 2 chain
DPBS	Dulbecco’s phosphate-buffered saline
ECM	extracellular matrix
FBS	fetal bovine serum
G′	storage modulus
G″	loss modulus
IBSP	integrin-binding sialoprotein
hBMSC	human bone marrow stem/stromal cell
HA	hydroxyapatite
LVR	linear viscoelastic region
MSC	mesenchymal stem/stromal cell
nHA	nano-hydroxyapatite
qPCR	quantitative real-time polymerase chain reaction
RUNX2	runt-related transcription factor 2
SPP1	secreted phosphoprotein-1
SE	standard error of the mean

## References

[1] Wu A.-M., Bisignano C., James S.L., Abady G.G., Abedi A., Abu-Gharbieh E., Alhassan R.K., Alipour V., Arabloo J., Asaad M. (2021). Global, regional, and national burden of bone fractures in 204 countries and territories, 1990–2019: A systematic analysis from the Global Burden of Disease Study 2019. Lancet Healthy Longev..

[2] Polinder S., Haagsma J., Panneman M., Scholten A., Brugmans M., Van Beeck E. (2016). The economic burden of injury: Health care and productivity costs of injuries in the Netherlands. Accid. Anal. Prev..

[3] Borgström F., Karlsson L., Ortsäter G., Norton N., Halbout P., Cooper C., Lorentzon M., McCloskey E.V., Harvey N.C., Javaid M.K. (2020). Fragility fractures in Europe: Burden, management and opportunities. Arch. Osteoporos..

[4] Caddeo S., Boffito M., Sartori S. (2017). Tissue Engineering Approaches in the Design of Healthy and Pathological In Vitro Tissue Models. Front. Bioeng. Biotechnol..

[5] Owen R., Reilly G.C. (2018). In vitro Models of Bone Remodelling and Associated Disorders. Front. Bioeng. Biotechnol..

[6] Kwakwa K.A., Vanderburgh J.P., Guelcher S.A., Sterling J.A. (2017). Engineering 3D Models of Tumors and Bone to Understand Tumor-Induced Bone Disease and Improve Treatments. Curr. Osteoporos. Rep..

[7] Tripathi S., Mandal S.S., Bauri S., Maiti P. (2023). 3D bioprinting and its innovative approach for biomedical applications. MedComm.

[8] Im S., Choe G., Seok J.M., Yeo S.J., Lee J.H., Kim W.D., Lee J.Y., Park S.A. (2022). An osteogenic bioink composed of alginate, cellulose nanofibrils, and polydopamine nanoparticles for 3D bioprinting and bone tissue engineering. Int. J. Biol. Macromol..

[9] Vanderburgh J.P., Guelcher S.A., Sterling J.A. (2018). 3D bone models to study the complex physical and cellular interactions between tumor and the bone microenvironment. J. Cell. Biochem..

[10] Gonzalez-Fernandez T., Sikorski P., Leach J.K. (2019). Bio-instructive materials for musculoskeletal regeneration. Acta Biomater..

[11] Gonzalez-Fernandez T., Tenorio A.J., Campbell K.T., Silva E.A., Leach J.K. (2021). Alginate-Based Bioinks for 3D Bioprinting and Fabrication of Anatomically Accurate Bone Grafts. Tissue Eng. Part A.

[12] Datta S. (2023). Advantage of Alginate Bioinks in Biofabrication for Various Tissue Engineering Applications. Int. J. Polym. Sci..

[13] Mahmud R.U., Rahman M.Z., Hashmi S. (2024). 13.13—Synthesis and characterization of nanocomposites for tissue engineering. Comprehensive Materials Processing.

[14] Hariyadi D.M., Islam N. (2020). Current Status of Alginate in Drug Delivery. Adv. Pharmacol. Pharm. Sci..

[15] Shams E., Barzad M.S., Mohamadnia S., Tavakoli O., Mehrdadfar A. (2022). A review on alginate-based bioinks, combination with other natural biomaterials and characteristics. J. Biomater. Appl..

[16] Sudipto D., Ranjit B., Jonali D., Leonel P. (2019). Importance of Alginate Bioink for 3D Bioprinting in Tissue Engineering and Regenerative Medicine. Alginates.

[17] Ma C., Du T., Niu X., Fan Y. (2022). Biomechanics and mechanobiology of the bone matrix. Bone Res..

[18] Bielajew B.J., Hu J.C., Athanasiou K.A. (2020). Collagen: Quantification, biomechanics and role of minor subtypes in cartilage. Nat. Rev. Mater..

[19] Li Z., Ruan C., Niu X. (2023). Collagen-based bioinks for regenerative medicine: Fabrication, application and prospective. Med. Nov. Technol. Devices.

[20] Li Y., Liu Y., Li R., Bai H., Zhu Z., Zhu L., Zhu C., Che Z., Liu H., Wang J. (2021). Collagen-based biomaterials for bone tissue engineering. Mater. Des..

[21] Sousa A.C., Biscaia S., Alvites R., Branquinho M., Lopes B., Sousa P., Valente J., Franco M., Santos J.D., Mendonça C. (2022). Assessment of 3D-Printed Polycaprolactone, Hydroxyapatite Nanoparticles and Diacrylate Poly(ethylene glycol) Scaffolds for Bone Regeneration. Pharmaceutics.

[22] Sancilio S., Gallorini M., Di Nisio C., Marsich E., Di Pietro R., Schweikl H., Cataldi A. (2018). Alginate/Hydroxyapatite-Based Nanocomposite Scaffolds for Bone Tissue Engineering Improve Dental Pulp Biomineralization and Differentiation. Stem Cells Int..

[23] Quinlan E., López-Noriega A., Thompson E., Kelly H.M., Cryan S.A., O’Brien F.J. (2015). Development of collagen-hydroxyapatite scaffolds incorporating PLGA and alginate microparticles for the controlled delivery of rhBMP-2 for bone tissue engineering. J. Control. Release.

[24] Soleymani S., Naghib S.M. (2023). 3D and 4D printing hydroxyapatite-based scaffolds for bone tissue engineering and regeneration. Heliyon.

[25] Melo P., Montalbano G., Fiorilli S., Vitale-Brovarone C. (2021). 3D Printing in Alginic Acid Bath of In-Situ Crosslinked Collagen Composite Scaffolds. Materials.

[26] Chen Y., Zhou Y., Wang C. (2022). Investigation of Collagen-Incorporated Sodium Alginate Bioprinting Hydrogel for Tissue Engineering. J. Compos. Sci..

[27] Hassani A., Khoshfetrat A.B., Rahbarghazi R., Sakai S. (2022). Collagen and nano-hydroxyapatite interactions in alginate-based microcapsule provide an appropriate osteogenic microenvironment for modular bone tissue formation. Carbohydr. Polym..

[28] Campos J.M., Sousa A.C., Caseiro A.R., Pedrosa S.S., Pinto P.O., Branquinho M.V., Amorim I., Santos J.D., Pereira T., Mendonça C.M. (2019). Dental pulp stem cells and Bonelike(^®^) for bone regeneration in ovine model. Regen. Biomater..

[29] Merimi M., El Majzoub R., Lagneaux L., Agha D., Fatima B., Meuleman N., Fahmi H., Lewalle P., Mohammad F.-K., Najar M. (2021). The Therapeutic Potential of Mesenchymal Stromal Cells for Regenerative Medicine: Current Knowledge and Future Understandings. Front. Cell Dev. Biol..

[30] Aleynik D., Charykova I., Rubtsova Y., Linkova D., Farafontova E., Egorikhina M. (2024). Specific Features of the Functional Activity of Human Adipose Stromal Cells in the Structure of a Partial Skin-Equivalent. Int. J. Mol. Sci..

[31] Sai B., Dai Y., Fan S., Wang F., Wang L., Li Z., Tang J., Wang L., Zhang X., Zheng L. (2019). Cancer-educated mesenchymal stem cells promote the survival of cancer cells at primary and distant metastatic sites via the expansion of bone marrow-derived-PMN-MDSCs. Cell Death Dis..

[32] Kemp K.C., Hows J., Donaldson C. (2005). Bone marrow-derived mesenchymal stem cells. Leuk. Lymphoma.

[33] Foresti R., Rossi S., Pinelli S., Alinovi R., Sciancalepore C., Delmonte N., Selleri S., Caffarra C., Raposio E., Macaluso G. (2020). In-vivo vascular application via ultra-fast bioprinting for future 5D personalised nanomedicine. Sci. Rep..

[34] Lai J., Wang M. (2023). Developments of additive manufacturing and 5D printing in tissue engineering. J. Mater. Res..

[35] Hassani A., Avci Ç.B., Kerdar S.N., Amini H., Amini M., Ahmadi M., Sakai S., Bagca B.G., Ozates N.P., Rahbarghazi R. (2022). Interaction of alginate with nano-hydroxyapatite-collagen using strontium provides suitable osteogenic platform. J. Nanobiotechnol..

[36] Ojeda E., García-Barrientos Á., Martínez de Cestafe N., Alonso J.M., Pérez-González R., Sáez-Martínez V. (2022). Nanometric Hydroxyapatite Particles as Active Ingredient for Bioinks: A Review. Macromol.

[37] Li N., Guo R., Zhang Z.J. (2021). Bioink Formulations for Bone Tissue Regeneration. Front. Bioeng. Biotechnol..

[38] Ferreira M.J.S.L.M. (2022). Integrating 3D Bioprinting and Pluripotent Stem Cell Technologies for Articular Cartilage Tissue Engineering. Ph.D. Thesis.

[39] Chen Z.-Y., Gao S., Zhou R.-B., Wang R.-D., Zhou F. (2022). Dual-crosslinked networks of superior stretchability and toughness polyacrylamide-carboxymethylcellulose hydrogel for delivery of alendronate. Mater. Des..

[40] Kajave N.S., Schmitt T., Nguyen T.U., Kishore V. (2020). Dual crosslinking strategy to generate mechanically viable cell-laden printable constructs using methacrylated collagen bioinks. Mater. Sci. Eng. C Mater. Biol. Appl..

[41] Shehzad A., Mukasheva F., Moazzam M., Sultanova D., Abdikhan B., Trifonov A., Akilbekova D. (2023). Dual-Crosslinking of Gelatin-Based Hydrogels: Promising Compositions for a 3D Printed Organotypic Bone Model. Bioengineering.

[42] Naranjo-Alcazar R., Bendix S., Groth T., Gallego Ferrer G. (2023). Research Progress in Enzymatically Cross-Linked Hydrogels as Injectable Systems for Bioprinting and Tissue Engineering. Gels.

[43] Bercea M. (2023). Rheology as a Tool for Fine-Tuning the Properties of Printable Bioinspired Gels. Molecules.

[44] Kostenko A., Connon C.J., Swioklo S. (2022). Storable Cell-Laden Alginate Based Bioinks for 3D Biofabrication. Bioengineering.

[45] Paar A. Amplitude Sweeps. https://wiki.anton-paar.com/uk-en/amplitude-sweeps/#:~:text=The%20limit%20of%20the%20linear,with%20the%20lowest%20strain%20values.

[46] Kocen R., Gasik M., Gantar A., Novak S. (2017). Viscoelastic behaviour of hydrogel-based composites for tissue engineering under mechanical load. Biomed. Mater..

[47] Shaabani A., Sedghi R., Motasadizadeh H., Dinarvand R. (2021). Self-healable conductive polyurethane with the body temperature-responsive shape memory for bone tissue engineering. Chem. Eng. J..

[48] Martinez A.W., Caves J.M., Ravi S., Li W., Chaikof E.L. (2014). Effects of crosslinking on the mechanical properties, drug release and cytocompatibility of protein polymers. Acta Biomater..

[49] Yi B., Xu Q., Liu W. (2022). An overview of substrate stiffness guided cellular response and its applications in tissue regeneration. Bioact. Mater..

[50] Sharma R., Kirsch R., Valente K.P., Perez M.R., Willerth S.M. (2021). Physical and Mechanical Characterization of Fibrin-Based Bioprinted Constructs Containing Drug-Releasing Microspheres for Neural Tissue Engineering Applications. Processes.

[51] Moeinzadeh S., Jabbari E. (2015). Gelation characteristics, physico-mechanical properties and degradation kinetics of micellar hydrogels. Eur. Polym. J..

[52] Rizwan M., Chan S.W., Comeau P.A., Willett T.L., Yim E.K.F. (2020). Effect of sterilization treatment on mechanical properties, biodegradation, bioactivity and printability of GelMA hydrogels. Biomed. Mater..

[53] Wu Z., Su X., Xu Y., Kong B., Sun W., Mi S. (2016). Bioprinting three-dimensional cell-laden tissue constructs with controllable degradation. Sci. Rep..

[54] Wei L., Li Z., Li J., Zhang Y., Yao B., Liu Y., Song W., Fu X., Wu X., Huang S. (2020). An approach for mechanical property optimization of cell-laden alginate-gelatin composite bioink with bioactive glass nanoparticles. J. Mater. Sci. Mater. Med..

[55] Mao Q., Wang Y., Li Y., Juengpanich S., Li W., Chen M., Yin J., Fu J., Cai X. (2020). Fabrication of liver microtissue with liver decellularized extracellular matrix (dECM) bioink by digital light processing (DLP) bioprinting. Mater. Sci. Eng. C.

[56] Liu W., Borrell M.A., Venerus D.C., Mieler W.F., Kang-Mieler J.J. (2019). Characterization of biodegradable microsphere-hydrogel ocular drug delivery system for controlled and extended release of ranibizumab. Transl. Vis. Sci. Technol..

[57] Li Z., Huang S., Liu Y., Yao B., Hu T., Shi H., Xie J., Fu X. (2018). Tuning Alginate-Gelatin Bioink Properties by Varying Solvent and Their Impact on Stem Cell Behavior. Sci. Rep..

[58] Noh I., Kim N., Tran H.N., Lee J., Lee C. (2019). 3D printable hyaluronic acid-based hydrogel for its potential application as a bioink in tissue engineering. Biomater. Res..

[59] Malektaj H., Drozdov A.D., deClaville Christiansen J. (2023). Swelling of Homogeneous Alginate Gels with Multi-Stimuli Sensitivity. Int. J. Mol. Sci..

[60] Naomi R., Ridzuan P.M., Bahari H. (2021). Current Insights into Collagen Type I. Polymers.

[61] Iviglia G., Cassinelli C., Torre E., Baino F., Morra M., Vitale-Brovarone C. (2016). Novel bioceramic-reinforced hydrogel for alveolar bone regeneration. Acta Biomater..

[62] Fischer L., Nosratlo M., Hast K., Karakaya E., Ströhlein N., Esser T.U., Gerum R., Richter S., Engel F.B., Detsch R. (2022). Calcium supplementation of bioinks reduces shear stress-induced cell damage during bioprinting. Biofabrication.

[63] Du L., Qin C., Zhang H., Han F., Xue J., Wang Y., Wu J., Xiao Y., Huan Z., Wu C. (2023). Multicellular Bioprinting of Biomimetic Inks for Tendon-to-Bone Regeneration. Adv. Sci..

[64] Fideles S.O.M., Ortiz A.C., Assis A.F., Duarte M.J., Oliveira F.S., Passos G.A., Beloti M.M., Rosa A.L. (2019). Effect of cell source and osteoblast differentiation on gene expression profiles of mesenchymal stem cells derived from bone marrow or adipose tissue. J. Cell. Biochem..

[65] Kriegel A., Schlosser C., Habeck T., Dahmen C., Götz H., Clauder F., Armbruster F.P., Baranowski A., Drees P., Rommens P.M. (2022). Bone Sialoprotein Immobilized in Collagen Type I Enhances Bone Regeneration In Vitro and In Vivo. Int. J. Bioprint..

[66] Kriegel A., Langendorf E., Kottmann V., Kämmerer P.W., Armbruster F.P., Wiesmann-Imilowski N., Baranowski A., Gercek E., Drees P., Rommens P.M. (2023). Bone Sialoprotein Immobilized in Collagen Type I Enhances Angiogenesis In Vitro and In Ovo. Polymers.

[67] Ogata Y. (2008). Bone sialoprotein and its transcriptional regulatory mechanism. J. Periodontal Res..

[68] Van der Heide D., Hatt L.P., Della Bella E., Hangartner A., Lackington W.A., Yuan H., De Groot-Barrère F., Stoddart M.J., D’Este M. (2024). Characterization and biological evaluation of 3D printed composite ink consisting of collagen, hyaluronic acid and calcium phosphate for bone regeneration. Carbohydr. Polym. Technol. Appl..

[69] Singh A., Gill G., Kaur H., Amhmed M., Jakhu H. (2018). Role of osteopontin in bone remodeling and orthodontic tooth movement: A review. Prog. Orthod..

[70] Liu B., Li J., Lei X., Cheng P., Song Y., Gao Y., Hu J., Wang C., Zhang S., Li D. (2020). 3D-bioprinted functional and biomimetic hydrogel scaffolds incorporated with nanosilicates to promote bone healing in rat calvarial defect model. Mater. Sci. Eng. C.

[71] Viguet-Carrin S., Garnero P., Delmas P.D. (2006). The role of collagen in bone strength. Osteoporos. Int..

[72] Gromolak S., Krawczenko A., Antończyk A., Buczak K., Kiełbowicz Z., Klimczak A. (2020). Biological Characteristics and Osteogenic Differentiation of Ovine Bone Marrow Derived Mesenchymal Stem Cells Stimulated with FGF-2 and BMP-2. Int. J. Mol. Sci..

[73] Strassburg S., Richardson S.M., Freemont A.J., Hoyland J.A. (2010). Co-culture induces mesenchymal stem cell differentiation and modulation of the degenerate human nucleus pulposus cell phenotype. Regen. Med..

[74] Senior J.J., Cooke M.E., Grover L.M., Smith A.M. (2019). Fabrication of Complex Hydrogel Structures Using Suspended Layer Additive Manufacturing (SLAM). Adv. Funct. Mater..

[75] Moxon S.R., Cooke M.E., Cox S.C., Snow M., Jeys L., Jones S.W., Smith A.M., Grover L.M. (2017). Suspended Manufacture of Biological Structures. Adv. Mater..

[76] Minogue B.M., Richardson S.M., Zeef L.A., Freemont A.J., Hoyland J.A. (2010). Characterization of the human nucleus pulposus cell phenotype and evaluation of novel marker gene expression to define adult stem cell differentiation. Arthritis Rheum..

[77] Minogue B.M., Richardson S.M., Zeef L.A., Freemont A.J., Hoyland J.A. (2010). Transcriptional profiling of bovine intervertebral disc cells: Implications for identification of normal and degenerate human intervertebral disc cell phenotypes. Arthritis Res. Ther..

[78] Livak K.J., Schmittgen T.D. (2001). Analysis of Relative Gene Expression Data Using Real-Time Quantitative PCR and the 2^−ΔΔCT^ Method. Methods.

